# Psychosocial distress among in-school adolescents in Mozambique: a cross-sectional study using the Global School-Based Health Survey data

**DOI:** 10.1186/s13034-020-00344-4

**Published:** 2020-09-26

**Authors:** Hubert Amu, Abdul-Aziz Seidu, Wonder Agbemavi, Bernard Owusu Afriyie, Bright Opoku Ahinkorah, Edward Kwabena Ameyaw, Kwaku Kissah-Korsah

**Affiliations:** 1 Department of Population and Behavioural Sciences, School of Public Health, University of Health and Allied Sciences, Hohoe, Ghana; 2 Department of Population and Health, University of Cape Coast, Cape Coast, Ghana; 3 College of Public Health, Medical and Veterinary Sciences, James Cook University, Townsville, QLD Australia; 4 School of Public Health, Faculty of Health, University of Technology Sydney, Sydney, Australia

**Keywords:** Adolescents, Psychosocial distress, Mental health, Mozambique, SDGs

## Abstract

**Background:**

Poor mental health remains the leading cause of disability, with considerable negative impacts in low- and middle-income countries. In this study, we examined the prevalence and correlates of psychosocial distress among in-school adolescents in Mozambique.

**Methods:**

This was a cross-sectional study of 1918 in-school adolescents, using data from the 2015 Mozambique Global School-Based Health Survey. Descriptive and inferential statistics were adopted in analysing the data. Statistical significance was set at p < 0.05.

**Results:**

The prevalence of psychosocial distress was 21.2% (24.1% females and 18.5% males). Older adolescents [AOR = 1.681, 95% CI = 1.233–2.292] had higher odds of experiencing psychosocial distress, compared with younger adolescents. In terms of sex, males [AOR = 0.755, 95% CI 0.601–0.950] had lower odds of experiencing psychosocial distress, compared with females. Adolescents who were bullied [AOR = 1.451, 95% CI 1.150–1.831], physically attacked [AOR = 1.802, 95% CI 1.404–2.313], and engaged in a physical fight [AOR = 1.376, 95% CI 1.070–1.769] were respectively more likely to experience psychosocial distress than those who did not. Conversely, adolescents who had close friends [AOR = 0.503, 95% CI 0.372–0.681] had lower odds of being psychosocially distressed than those who did not have close friends.

**Conclusion:**

The prevalence of psychosocial distress among in-school adolescents in Mozambique is relatively high. The country may not be able to meet the Sustainable Development Goal 3.4 target of promoting mental health and wellbeing of all by the year 2030 if current rates of psychosocial distress persist among in-school adolescents. Mental health education and counselling as well as social support from friends should be intensified to reduce mental health problems and enable adolescents to effectively deal with the psychosocial challenges encountered in their transition from childhood to adulthood.

## Background

The definition of health by the World Health Organisation [1] as “the state of complete physical, mental and social wellbeing and not merely the absence of infirmity,” has entreated many low- and middle-income countries (LMICs) to improve the health of their populace. Despite several interventions and global agenda such as the Sustainable Development Goals (SDGs), mental health continues to remain one of the major public health problems in LMICs [2].

Poor mental health is the leading cause of disability, with considerable negative impacts in LMICs [3]. According to the WHO [3], mental health conditions account for 16% of the burden of disease and injury in people aged 10–19 years. The 2018 World Drug Report stated that half of all mental health conditions start by 14 years of age, but most cases are undetected and untreated [4]. Most often, signs of poor mental health are overlooked for several reasons, such as a lack of knowledge or awareness about mental health among health workers, or stigma which even prevents adolescents from seeking help [4].

The most common mental disorders in sub-Saharan Africa (SSA) are depression and anxiety [5, 6]. The prevalence of depression and major depressive disorder in the sub-region ranges from 40% to 55% [7]. Among the child and adolescent populations, mental health issues are common. Fourteen percent have mental health problems and nearly 10% have diagnosable psychiatric disorders [7].

In Mozambique, limited priority is given to the prevention, care, and treatment of mental health disorders, in comparison with other health conditions [8]. As of 2011, only 16% of the total health budget for the country was allocated toward mental health services [9]. This neglect of service provision happens at a period when mental, neurological, and substance use disorders are considered as the primary drivers of disability worldwide. According to the World Health Organization’s Assessment Instruments for Mental Health Systems (WHO-AIMS), the most common outpatient psychiatric consultations in Mozambique are for epilepsy (53%), child mental disorders (15%), and schizophrenia (14%) [8].

One of the commonest mental health conditions among adolescents is the development of psychosocial distress [10]. This is because, at this age, they are generally exposed to strain and stress due to several problems such as meeting the expectations for sexual and social relationships, homework, and examination [10]. These expectations incline them toward psychosocial distress. Psychosocial distress is an emotional state or mood characterised by the feeling of loneliness, sadness, anxiety, suicidal ideation, and self-consciousness [11]. Suicide, for instance, is the third leading cause of death in adolescents 15–19 years old [12].

Several factors have been identified to be associated with psychosocial distress among adolescents. Data from the US National Longitudinal Study of Adolescent Health found that single and social-parent family structure is adversely associated with emotional adjustment, depression, adolescent delinquency, cognitive skills, school engagement, school problems, and grade point average [13–15]. Individual and socio-cultural factors such as female sex, lower formal education and lower socio-economic status, lack of social support, and stressful life events have also shown to be important contributors [16].

In SSA, studies have been conducted to examine the potential correlates of psychosocial distress among in-school adolescents in Tanzania [17, 18] and Zambia [19]. Currently, there is a paucity of empirical literature on psychosocial distress among in**-**school adolescents in Mozambique. This is essential because a study has identified children and adolescents in Mozambique as the cohort with high odds of mental disorders, including psychosocial distress [20]. Due to this, the present study examined the prevalence and correlates of psychosocial distress among adolescents in Mozambique. The study is essential in contributing to the knowledge base and would inform interventions needed to reduce psychosocial distress among in-school adolescents in Mozambique. Potential correlates identified in this study will help Government and non-governmental organisations focused on improving mental health in Mozambique to channel the majority of their resources to the major factors predisposing in-school adolescents to psychosocial distress.

## Methods

### Data source

This study was cross-sectional and used data from the 2015 Mozambique Global School-Based Health Survey (GSHS) collected using a clustered sample design. The Mozambique GSHS was conducted in 2015 by WHO in collaboration with the United Nations Children’s Fund (UNICEF), United Nations Educational, Scientific and Cultural Organization (UNESCO), and The Joint United Nations Programme on HIV and AIDS (UNAIDS) with technical assistance from the Centres for Disease Control and Prevention (CDC) [21]. The GSHS aims to provide data on health and social behaviours among in-school adolescents. The Mozambique GSHS was a school-based survey of students in Class 8–12. A two-stage cluster sample design was used to produce data that are representative of all students in class 8–12 in Mozambique.

The initial stage of the sampling was characterised by the selection of schools with probabilities proportional to enrolment size. This was followed by a random selection of classes and all students in selected classes. The Mozambique GSHS measured alcohol use, dietary behaviours, drug use, hygiene, mental health, physical activity, sexual behaviours, tobacco use, violence and unintentional injury. The students answered the survey questionnaires on a computer scannable answer sheet. The school response rate was 97%, the student response rate was 83%, and the overall response rate was 80% [21]. A total of 1918 students participated in the survey. Before commencement of the GSHS, permission to conduct the study was obtained from the Ministries of Health and Education. Informed consent to participate in the study was obtained from school managers and students. Students anonymously and voluntarily completed the questionnaire.

### Study variables

#### Outcome variable

The outcome variable “psychosocial distress” was assessed using five mental health indicators—loneliness, anxiety, suicidal ideation, suicidal attempt, and suicide plan—based on previous research on a similar in-school adolescent population [19, 21–24]. The original responses of these variables were transformed into dichotomous responses where 0 = no and 1 = yes (see Table 1). In deriving the outcome variable, adolescents who reported two or more of these five mental health indicators were coded as 1, denoting psychosocial distress whilst those who had less than two of the indicators were coded otherwise ‘0’ following categorisation from previous studies [19].

**Table 1:** Description of the study variables

Variables	Question	Response options and recoding
Outcome variable		
Psychosocial distress	Assessed using three mental health measures	0–5 (Coded as 1 = 2–5 and 0 = < 2)
Loneliness	During the past 12 months, how often have you felt lonely?	1 = never, 2 = rarely, 3 = sometimes, 4 = most of the time to 5 = always (coded as 1–3 = 0 and 4- 5 = 1)
Anxiety	During the past 12 months, how often have you been so worried about something that you could not sleep at night?	1 = never to 5 = always (coded 1–3 = 0 and 4 – 5 = 1)
Suicide plan	During the past 12 months, did you make a plan about how you would attempt suicide?	1 = Yes 2 = No Coded (1 = Yes and 0 = No)
Suicidal attempt		1 = Yes 2 = No Coded (1 = Yes and 0 = No)
Suicidal ideation		
Explanatory variables		
Age	Custom age	1 = 12, 2 = 13, 3 = 14, 4 = 15, 5 = 16, 6 = 17, 7 = 18 years (coded as 0 = 12–14, 15 +=1)
Sex	Sex	1 = male, 2 = female
Grade	In what grade are you?	1 = 8th
		2 = 9th
		3 = 10th
		4 = 11th
		5 = 12th
Hunger (proxy of socioeconomic status)	Went hungry past 30 days	1 = never, 2 = rarely, 3 = sometimes, 4 = most of the times, 5 = always (coded 1–3 = 0, 4–5 = 1)
Alcohol use	During the past 30 days, on how many days did you have at least one drink containing alcohol?	1 = 0 days; to 7 = All 30 days (coded as 1 = 0; and 2–7 = 1)
Smoking	During the past 30 days, how many days did you smoke cigarette?	1 = 0 days; to 7 = All 30 days (coded as 1 = 0; and 2–7 = 1)
Fight	During the past 12 months, how many times were you in a physical fight?	1 = 0 times; to 8 = 12 or more times (coded 1 = 0; and 2–8 = 1)
Bullied	During the past 30 days, how were you bullied most often?	1 = 0 times; to 8 = 12 or more times (coded 1 = 0; and 2–7 = 1)
Close friends	How many close friends do you have?	1 = 0 to 4 = 3 or more (coded as 1 = 0, 1–2 = 1, 3 or more = 2)
Truancy	During the past 30 days, on how many days did you miss classes or school without permission?	1 = 0 days,2 = 1or 2 days, 3 = 3 to 5 days, 4 = 6 to 9 days, 5 = 10 or more (coded 1 = 0 and 2–5 = 1)
Attacked	During the past 12 months, how many times were you physically attacked?	1 = 0 days,2 = 1or 2 days, 3 = 3 to 5 days, 4 = 6 to 9 days, 5 = 10 or more (coded 1 = 0 and 2–5 = 1)
Physical Activity	During the past 7 days, on how many days were you physically active for a total of at least 60 min per day?	﻿1 = 0 days to 8 = all 7 days (coded 1 = 5 or more days and 0–4 days = 0)
Helpful (Peer support)	During the past 30 days, how often were most of the students in your school kind and helpful?	1 = never, 2 = rarely, 3 = sometimes, 4 = most of the times, 5 = always (coded 1–3 = 0; and 4–5 = 1)
Parents check homework (parental supervision)	During the past 30 days, how often did your parents or guardians check to see if your homework was done?	1 = never, 2 = rarely, 3 = sometimes, 4 = most of the times, 5 = always (coded 1–3 = 0; and 4–5 = 1)
Parents understand problems and worries (Parental Connectedness)	During the past 30 days, how often did your parents or guardians understand your problems and worries?	1 = never, 2 = rarely, 3 = sometimes, 4 = most of the times, 5 = always (coded 1–3 = 0; and 4–5 = 1)
Parents know what adolescent do free time (Parental or guardian Bonding)	During the past 30 days, how often did your parents or guardians really know what you were doing with your free time?	1 = never, 2 = rarely, 3 = sometimes, 4 = most of the times, 5 = always (coded 1–3 = 0; and 4–5 = 1)

### Explanatory variables

Sixteen explanatory variables were included in the estimations. They were sex, age, grade, alcohol use, bullied, physically attacked, truancy, ever smoked, the number of close friends, engaged in a fight, parental or guardian supervision, getting help from friends, physical activity, parental connectedness, experience of hunger, and parental bonding. The coding and selection of these variables were based on previous studies [11, 19, 23, 24] and their availability in the GSHS dataset. Detailed descriptions of the variables and coding are presented in Table 1.

### Statistical analysis

The 2015 Mozambique GSHS dataset has more than 5% of values that are missing at random (MAR) pattern. To account for the consistency of the dedicated sample size values of each variable throughout the analyses, we adopted a multiple imputation method to handle the missing values [25, 26]. A full conditional specification method was used after automatic command scanned for the data which were missing at random. A maximum of five imputations was run to allow for > 97% efficiency [26]. Data analyses were performed using STATA version 14.2 software for Mac OS. Due to the nature of the study design, a weighting factor was used in the analysis to reflect the likelihood of sampling each pupil and to reduce bias by compensating for differing patterns of nonresponse. A descriptive analysis was done to describe the general characteristics of the study population and the prevalence of psychosocial distress and reported p-values of Pearson’s Chi Square (bivariate analysis).

The explanatory variables with *p* value less than 0.05 (statistically significant) were included in the multivariate analysis. The variables which were statistically significant in the multivariable analysis were then discussed. Multicollinearity was checked with the Variance Inflation Factor (Mean VIF = 1.06, Maximum VIF = 1.17, Minimum VIF = 1.02). The multivariable analysis in the form of a binary logistic regression model was used to determine the strength of association between the explanatory and the outcome variable. The results from the regression analyses were presented as adjusted odds ratios (AOR). The choice of reference categories for all the explanatory variables was informed by previous studies [11, 19, 23, 24].

## Results

### Descriptive results

Table 2 presents the background characteristics and the prevalence of psychosocial distress among in-school adolescents. The prevalence of psychosocial distress was 21.2% (24.1% female and 18.5% male). It was higher among older adolescents (23.4%) than younger ones (15.8%). Adolescents who reported being hungry (28.1%), those who were bullied (28.6%), those who engaged in a physical fight (26.9%), those who smoked (34.4%), and those who were physically attacked (29.2%) respectively reported higher proportions of psychosocial distress. Similarly, adolescents who did not have close friends (37.1%) and those who were truant reported higher proportions of psychosocial distress. Our Chi square analysis showed that age, sex, hunger, being bullied, engagement in a physical fight, smoking, being physically attacked, engagement in physical activity, truancy, having close friends, having peer support, and parents’ understanding of problems and worries showed statistically significant associations with psychosocial distress (Table 2).

**Table 2:** Background characteristics and prevalence of psychosocial distress among in-school adolescents in Mozambique

Variables	N = 1918		Psychosocial distress		Chi square (X^2^) (p-value)
	Frequency	Percentage (%)	No (78.8%)	Yes (21.2%)	
Age					5.9 (0.016)
11–14	560	29.2	84.16	15.84	
15–18	1358	70.8	76.58	23.42	
Sex					4.9 (0.034)
Female	938	48.89	75.95	24.05	
Male	980	51.11	81.51	18.49	
Grade					4.9 (0.300)
8th class	569	29.65	79.90	20.10	
9th class	472	24.61	81.67	18.33	
10th class	517	26.96	76.41	23.59	
11th class	199	10.36	77.67	22.33	
12th class	162	8.42	75.49	24.51	
Hungry					12.5 (< 0.001)
No	1708	89.07	79.64	20.36	
Yes	210	10.93	71.91	28.09	
Bullied					26.1 (< 0.001)
No	1145	59.70	83.81	16.19	
Yes	773	40.30	71.37	28.63	
Physical fight					33.0(< 0.001)
No	1277	66.59	81.63	18.37	
Yes	641	33.41	73.13	26.87	
Alcohol					0.01 (0.929)
No	1716	89.46	79.25	20.75	
Yes	202	10.54	74.94	25.06	
Smoking					5.2 (0.023)
No	1868	97.42	79.14	20.86	
Yes	50	2.58	65.56	34.44	
Physically Attacked					48.6 (< 0.001)
No	1259	65.62	82.99	17.01	
Yes	659	34.38	70.78	29.22	
Physical activity					4.8 (0.028)
Less than 4 days	1523	79.40	79.48	20.52	
4+	395	20.60	76.14	23.86	
Truancy					6.6 (0.010)
No	1456	75.93	80.63	19.37	
Yes	462	24.07	73.00	27.00	
Close Friends					28.5 (< 0.001)
0	291	15.2	62.87	37.13	
1+	1627	84.8	81.64	18.36	
Peers support					6.2 (0.013)
No	1308	68.21	77.69	22.31	
Yes	610	31.79	81.15	18.85	
Parents checking Homework					0.1 (0.725)
No	1032	53.79	78.23	21.77	
Yes	886	46.21	79.45	20.55	
Parents’ understanding of problems and worries					4.8 (0.028)
No	1052	54.86	78.09	21.91	
Yes	866	45.14	79.65	20.35	
Free-time					0.087 (0.767)
No	1182	61.61	78.88	21.12	
Yes	736	38.39	78.65	21.35	

Table 3 presents results on the multivariable logistic regression analysis of the correlates of psychosocial distress. Older adolescents [AOR = 1.681, 95% CI 1.233–2.292] were more likely to experience psychosocial distress than younger adolescents. Adolescents who experienced hunger [AOR = 1.614, 95% CI 1.157–2.250], were bullied [AOR = 1.451, 95% CI 1.150–1.831], physically attacked [AOR = 1.802, 95% CI 1.404–2.313], and those who engaged in a physical fight [AOR = 1.376, 95% CI 1.070–1.769] were respectively more likely to experience psychosocial distress than those who did not. Conversely, males [AOR = 0.755, 95% CI 0.601–0.950] and adolescents who had close friends [AOR = 0.503, 95% CI 0.372–0.681] were respectively less likely to be psychosocially distressed than females and those who did not have close friends (Table 3).

**Table 3:** Logistic regression analyses on the correlates of psychosocial distress among in-school adolescents in Mozambique

Variables	AOR [95% CI]
Age	
11–14	Ref
15–18	1.681**[1.233–2.292]
Sex	
Female	Ref
Male	0.755* [0.601–0.950]
Hunger	
No	Ref
Yes	1.614** [1.157–2.250]
Bullied	
No	Ref
Yes	1.451** [1.150–1.831]
Physical fight	
No	Ref
Yes	1.376* [1.070–1.769]
Smoking	
No	Ref
Yes	1.205 [0.658–2.208]
Physically attacked	
No	Ref
Yes	1.802*** [1.404–2.313]
Physical activity	
Less than 4 days	Ref
4+	1.241 [0.942–1.635]
Truant	
No	Ref
Yes	1.126 [0.874–1.450]
Number of close friends	
0	Ref
1+	0.503*** [0.372–0.681]
Peers support	
No	Ref
Yes	0.838 [0.648–1.084]
Parents’ understanding of problems and worries	
No	Ref
Yes	0.876 [0.691–1.110]
N	1918
Pseudo R^2^	0.062

^*^p < 0.05, ^**^p < 0.01, ^***^p < 0.001; AOR, Adjusted Odds Ratio; Ref, Reference

## Discussion

This study examined the prevalence and correlates of psychosocial distress in Mozambique using the 2015 Mozambique GSHS data on in-school adolescents. The prevalence of psychosocial distress was 21.2%. Older adolescents, those who went hungry, those who were bullied, those who engaged in a physical fight, and adolescents who were physically attacked recorded significantly higher probabilities of being psychosocially distressed. On the other hand, males and adolescents who had close friends were less likely to experience psychosocial distress.

The prevalence of psychosocial distress observed in the present study was higher than those reported by Siziya and Mazaba [19] (15.7%), Seidu et al. [24] (16.9%), and Olga et al. [27] (10.8%) in other SSA countries. The comparatively higher prevalence of psychosocial distress found in our study reflects the relatively higher mental health problems among adolescents in Mozambique. For instance, while Seidu et al. [28] reported the prevalence of suicidal attempt among in-school adolescents in Mozambique as 18.5%, Amare et al. [29] and Omigbodun [30] found 16.2% and 12% respectively in Ethiopia and Nigeria. The variations in the prevalence of psychosocial distress between our findings and the other studies could be due to differences in socio-political situations prevalent in the respective countries, which have been shown to have implications for the general mental health of pupils in school environments [31, 32]. For instance, while Ghana, Zambia, and Benin have enjoyed over a decade of stability, socio-political conflicts in Mozambique have witnessed renewed conflicts leading to the assassination of the academic constitutional lawyer, Gilles Cistac, in March 2015 [33] as well as the current insurgency in Cabo Delgado Province [34].

The comparatively high prevalence of psychosocial distress found in our study comes at the backdrop that as a country, Mozambique is a signatory to the SDG target of promoting mental health and wellbeing for all by the year 2030 [35]. Promulgated in the year 2015, the SDGs are 17 global goals with 169 targets that all United Nations member countries have decided to work towards achieving by the year 2030 [36, 37]. The SDG 3 seeks to ensure healthy lives and promote wellbeing for all at all ages and the fourth (target 3.4) out of 13 targets under goal three is what seeks to address mental health. The high prevalence of psychosocial distress observed in our study could, therefore, inhibit the country’s efforts at achieving the SDG target.

We found that age served as an important correlate of psychosocial distress among in-school adolescents. Older adolescents experienced significantly higher proportions and odds of psychosocial distress than the younger ones. This corroborates findings of previous studies which argued that older adolescents experience higher rates of mental health challenges than younger ones [38, 39]. The findings may be attributed to the fact that the major transitions from childhood to adulthood begin from late adolescence, and this becomes very stressful for the young people as they try to become more self-sufficient and take decisions that shape and inform their future [40]. Decisions such as making educational plans, finding their accommodations, starting serious sexual relationships, and starting careers are usually taken at this stage [40].

Sex was also an important correlate of psychosocial distress in our study. We found that male adolescents were less likely to experience psychosocial distress than females. This observation could be because female adolescents are more prone to sexual abuse and harassment or exploitation which have negative implications for psychosocial distress [16, 41]. Besides, the lower odds of psychological distress observed among males could be explained by the fact that male adolescents are more likely to form peer groups and are also more likely to confide in their friends, which helps them to mitigate the emotional problems which characterise adolescence [42, 43].

Being bullied, engagement in a physical fight, being physically attacked, and going hungry served as factors which increased the odds of experiencing psychosocial distress in our study. Bullying has, for instance, been established as one of the persistent difficulties in schools and it has negative consequences for the physical, social, and mental health of students such as suicidal ideation and depression [44–46]. Having close friends, however, decreased the odds of experiencing psychosocial distress among in-school adolescents in our study. Our findings point to the role of social support in ameliorating the effects of mental health challenges which adolescents face as part of the vicissitudes involved in the transition from childhood to adulthood [47–52].

## Strengths and limitations

The study made use of a dataset with a relatively large sample which gave the study the statistical power to run a rigorous analysis. ﻿Despite the important findings made and the rigour involved in our analyses, the results should be interpreted with these limitations in mind. First, our study shares all the shortfalls associated with cross-sectional study design, most especially regarding establishing causality. Secondly, there is the possibility of recall and social desirability biases from adolescents. Thirdly, the fact that our study focused on only in-school adolescents means the results are not generalizable to all adolescents in the country [26]. Moreover, since the study used secondary data (GSHS), information on other variables such as socioeconomic status, religious affiliation, social participation, and psychological co-morbidities that could be important in characterizing psychosocial distress could not be assessed [53]. Some of the studies that our results were compared with at the discussion section used other assessment tools and not that of the global school-based health survey.

## Conclusion

Psychosocial distress is relatively high among in-school adolescents in Mozambique. Older adolescents experience higher proportions and probabilities of psychosocial distress. Mozambique may not be able to meet the SDG 3.4 target of promoting mental health and wellbeing by the year 2030 if current rates of psychosocial distress persist. For Mozambique to accelerate progress towards meeting the SDG targets of promoting mental health and wellbeing as well as ending hunger by the year 2030, we recommend that mental health education and counselling be intensified by the Ministry of Health and other stakeholders in schools. Increased social support from family, peers, and friends is recommended to enable adolescents to effectively deal with the psychosocial challenges they are confronted with at this transition phase of their life.

## Data Availability

The datasets analysed during the current study are available at https://www.who.int/ncds/surveillance/gshs/mozambiquedataset/en/.

## Abbreviations

CDC: Centres for Diseases Control and Prevention
GSHS: Global School-Based Health Survey
UNICEF: United Nations Children’s Fund
WHO: SDG: Sustainable Development Goal, World Health Organisation
